# A Holistic View of Psoriasis: Examining Its Association With Dyslipidemia and Obesity in a Decade-Long Systematic Review and Meta-Analysis

**DOI:** 10.7759/cureus.49241

**Published:** 2023-11-22

**Authors:** Jaber Abdullah Alshahrani, Alaa Mohammed Alshahrani, Shahad Ali Alshahrani, Fatimah Abdullah Alshahrani, Matar Saeed Matar Alzahrani, Rima Jaza Albalawi, Mohammed A Aljunaid

**Affiliations:** 1 Family Medicine and Medical Education Department, Armed Forces Hospitals Southern Region, Khamis Mushait, SAU; 2 Family Medicine Department, Armed Forces Hospitals Southern Region, Khamis Mushait, SAU; 3 College of Pharmacy, King Khalid University, Abha, SAU; 4 Abha Health Sector, Ministry of Health, Abha, SAU; 5 Family Medicine, Alhajrah General Hospital, Ministry of Health, Alhajrah, SAU; 6 College of Medicine, University of Tabuk, Tabuk, SAU; 7 Family and Community Medicine Department, University of Jeddah, Jeddah, SAU

**Keywords:** systematic review and meta-analysis, derma, eczema, lipid, dermatology, systematic review, meta-analysis, psoriasis, obesity, dyslipidemia

## Abstract

Psoriasis is a multifaceted inflammatory condition with systemic implications, impacting not only the skin but also various organs and overall health. It is associated with mood disorders, malignancy, infections, and components of metabolic syndrome, including diabetes, atherogenic dyslipidemia, and obesity. The coexistence of psoriasis with obesity poses additional challenges, as obesity worsens psoriasis severity and reduces treatment effectiveness. This systematic review and meta-analysis aim to further understand the associations between psoriasis, dyslipidemia, and obesity. Our systematic review of six studies revealed significant links between psoriasis and both dyslipidemia and obesity. Individuals with psoriasis exhibited a 1.40-fold higher likelihood of dyslipidemia (odds ratio (OR) 1.40, 95% confidence interval (CI) 1.24-1.58) and a 1.37-fold higher likelihood of obesity (OR 1.37, 95% CI 1.23-1.53) compared to those without psoriasis. These findings emphasize the systemic nature of psoriasis and its implications for metabolic health. In conclusion, this review underscores the importance of holistic management for psoriasis patients. Further research is warranted to explore underlying mechanisms and develop targeted therapeutic strategies. These findings contribute valuable insights to promote the overall well-being of individuals with psoriasis.

## Introduction and background

Psoriasis, a chronic and multifaceted inflammatory disorder, extends its impact far beyond the skin's boundaries, affecting multiple organ systems, including the cardiovascular, renal, and gastrointestinal systems [1]. Beyond its physical manifestations, this condition has been linked to mood disorders, malignancies, and infections, emphasizing the systemic nature of its influence [1]. Psoriasis's immunological underpinnings connect it with various components of the metabolic syndrome, encompassing diabetes mellitus, insulin resistance, atherogenic dyslipidemia, hypertension, central adiposity, and metabolic-associated fatty liver disease [2].

The prevalence of metabolic syndrome among psoriasis patients varies, with estimates ranging from 20% to 50%, contingent on disease severity [3]. This association between psoriasis and metabolic syndrome is not merely coincidental; instead, it underscores shared metabolic pathways, genetic factors, and pathogenic mechanisms [1]. Consequently, the chronic systemic use of psoriasis treatments necessitates cautious consideration to prevent exacerbating coexisting metabolic conditions. In parallel, the global surge in obesity rates, currently afflicting approximately one-third of the world's population [4], compounds the health challenges faced by individuals with psoriasis.

Recognizing this, patients with psoriasis emerge as prime candidates for targeted obesity screening and weight reduction strategies to mitigate the potential health consequences. Existing literature underscores the therapeutic benefits of weight reduction in ameliorating psoriasis severity [5]. Notably, an elevated body mass index emerges as a detriment to the efficacy of biological therapies in managing psoriasis [6]. Beyond obesity, dyslipidemia, characterized by altered lipid profiles, has surfaced as another crucial facet of the psoriasis landscape.

Several studies have illuminated the association between psoriasis and dyslipidemias, with Salihbegovic et al. (2015) elucidating links between psoriasis and hypertriglyceridemia and high-density lipoproteins [7]. These findings corroborate the work of Nakhwa et al. (2014) and prompt explorations into the underlying mechanisms [8]. Plausible explanations encompass insufficient physical activity, dietary habits, and the proinflammatory effects of cytokines on lipid metabolism [9]. Furthermore, the treatment of psoriasis with agents, such as cyclosporine and acitretin, may contribute to secondary dyslipidemias [10]. Given the grave implications of dyslipidemia for cardiovascular health, investigating the intricate interplay between dyslipidemia and psoriasis emerges as a critical pursuit. Therefore, this systematic review and meta-analysis seeks to comprehensively evaluate the intricate relationships between psoriasis, dyslipidemia, and obesity, shedding light on their clinical implications and providing valuable insights for tailored patient management strategies.

## Review

Materials and methods

This section is dedicated to elucidating the research methodology, specifically focusing on our search strategy, selection process, data items, effect measures, and methods for data collection and synthesis.

Research Question

Our research question centers on understanding the extent to which psoriasis is associated with dyslipidemia and obesity.

Search Strategy

To construct a robust and thorough dataset for our meta-analysis, we employed a multi-faceted, algorithmically optimized search strategy. This methodical approach involved interrogating a diverse array of scholarly databases, including PubMed, MEDLINE, Scopus, Web of Science, and the Cochrane Library, as well as Google Scholar. The time window for the search was strategically chosen to span from January 2012 to July 2023, a period that maintains the study's currency while distancing it from previous work to ensure novelty.

Query Formulation

Our query leveraged a meticulously crafted Boolean logic, incorporating Medical Subject Headings (MeSH) terms and free-text keywords. The terms were selected based on their semantic richness and relevance to our research question. Our query comprised terms, such as "psoriasis," "dyslipidemia," "hyperlipidemia," "atherogenic lipids," "obesity," "BMI," and "metabolic syndrome."

Language and Secondary Sources

Given the global influence of English-language scientific literature, we confined our search to English-language articles to uphold data quality and streamline the extraction process. To bolster the comprehensiveness of our dataset, we also performed backward and forward citation chaining on articles identified in the initial database search. This practice ensured that no seminal work was missed and that we incorporated the most recent research developments.

Selection Process

The selection criteria for our meta-analysis were meticulously designed to achieve a high level of both internal and external validity. This process was subjected to a two-tiered review mechanism, in which each paper was independently evaluated by two trained reviewers for quality and relevance. In situations where discrepancies emerged between the reviewers, a third expert served as an arbitrator, facilitating a consensus decision.

Study Types and Design

We considered an array of study designs, including but not limited to cross-sectional studies, case-control studies, and both retrospective and prospective cohort studies. Due to the limitations of qualitative data in answering our research question, qualitative studies were intentionally excluded from this review.

Accessibility and Language Criteria

Both open- and closed-access articles were considered to minimize selection bias. However, we acknowledge that the inability to access some closed-access studies could potentially limit the review’s comprehensiveness. To ensure methodological rigor, we confined our selection to studies published in the English language.

Role of Librarian

A specialized research librarian was consulted during the study design phase to construct an exhaustive and precise search strategy. The librarian’s role was particularly valuable in refining our keyword list and Boolean query, thus ensuring a more comprehensive search result.

Exclusion Criteria

**Figure 1:**
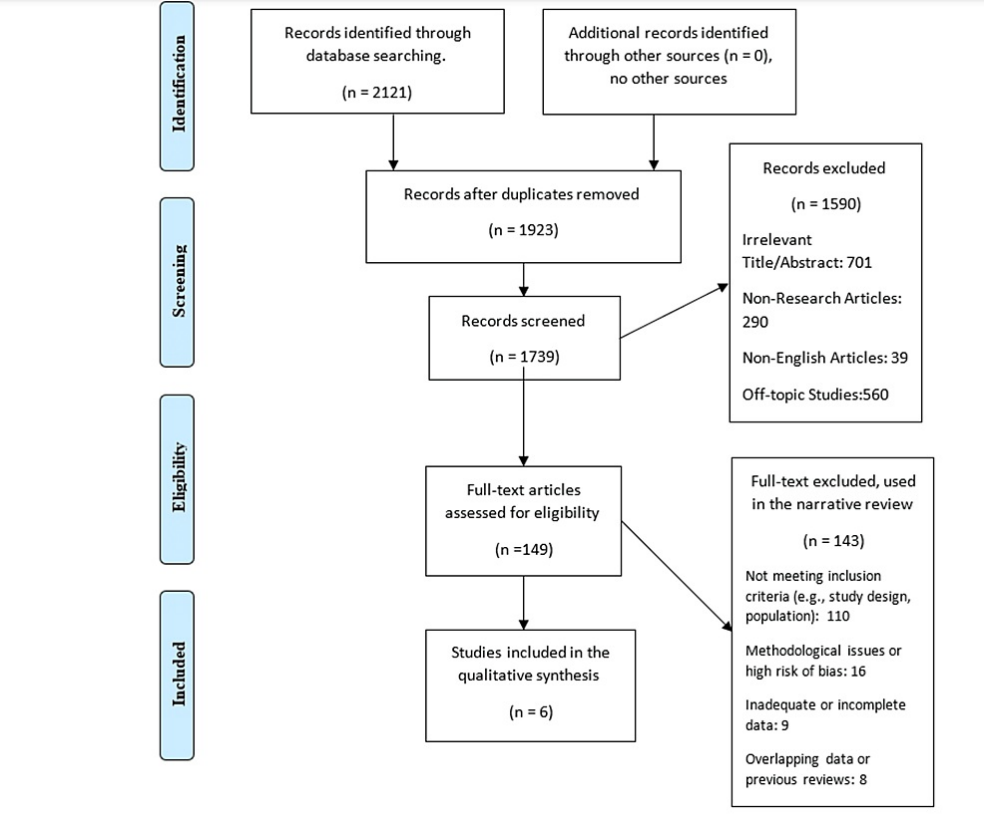
Dyslipidemia and obesity among patients with psoriasis and control subjects

To sustain the study's focus, we excluded case reports, case series, animal studies, and opinion pieces (Figure 1).

Data Items

The core data items extracted from the selected studies encompassed various aspects critical to our research:

Study characteristics: We collected detailed information about each study, including the author's name, publication year, country of publication, and study design. These details allowed us to contextualize the findings within each study.

Participants: We documented the total number of participants in both the psoriasis and control groups to understand the scale of each investigation.

Outcome Data: The primary outcome data consisted of the number of events (cases) of dyslipidemia and obesity observed within both the psoriasis and control groups. This information provided the foundation for assessing the association between psoriasis and these health outcomes.

Effect measures: We extracted the reported odds ratios (Ors) and their corresponding 95% confidence intervals (CIs) from each included study.

Effect Measures and Statistical Methods

Our study employed a range of effect measures to comprehensively understand the multidimensional relationship between psoriasis, dyslipidemia, and obesity. The primary effect measure was ORs, accompanied by their 95% CIs, which provided a robust quantitative method for evaluating the magnitude and direction of the associations. However, recognizing that not all studies report OR, secondary measures, such as relative risk (RR) and hazard ratios (HRs), were also considered where available.

Methodologically, the choice of OR as the primary effect measure was validated by ensuring its appropriateness for the types of studies included in our meta-analysis. For studies that did not report OR, we applied a statistical transformation to calculate the estimated OR from available measures, such as RR.

Data Collection

The process of data extraction was carried out meticulously by two independent reviewers to ensure accuracy and consistency. In cases of discrepancies or disagreements, a third reviewer was consulted to reach a consensus. A standardized data extraction form facilitated the systematic collection of information from each selected study, encompassing key elements, such as authorship, publication year, country of origin, prevalence of dyslipidemia and obesity among cases and controls, and participant demographics, including age and gender.

Risk-of-Bias Assessment

In an endeavor to fortify the methodological integrity and reliability of our meta-analysis, we conducted an exhaustive risk of bias assessment. We employed the Newcastle-Ottawa Scale (NOS), a tool that has received broad academic endorsement for its efficacy in evaluating the quality of non-randomized studies [11]. The NOS considers a triad of crucial dimensions: study selection, comparability of study groups, and the ascertainment of outcomes. Each study was rigorously scored on these domains, with scores in our assessment ranging between 7 and 8, indicative of high methodological quality. It is pivotal to note that the application of NOS in our meta-analysis was not just a procedural formality; rather, it served as a foundational measure to diminish the risk of bias and fortify the methodological rigor. By systematically applying NOS criteria, we ensured the inclusion of studies that met high methodological standards, thereby augmenting the robustness of our findings. This assiduous approach underpins the validity and reliability of our investigative conclusions on the relationships between psoriasis, dyslipidemia, and obesity.

Statistical Analysis

For the purpose of conducting a methodologically rigorous meta-analysis, we utilized the Review Manager (RevMan) software, a specialized computational framework developed by the Cochrane Collaboration for the analysis of complex healthcare data. Employing the DerSimonian and Laird random-effects model, we estimated the pooled ORs along with their corresponding 95% CIs. This model is particularly well suited for meta-analyses involving observational studies, as it accommodates between-study heterogeneity, thereby providing a more robust and reliable pooled estimate (DerSimonian & Laird, 1986).

Given that the studies included in our meta-analysis were observational in nature, we opted for random-effects models over fixed-effects models. This decision acknowledges the inherent diversity among the study populations and methodologies. Data for dichotomous outcomes were meticulously entered by hand to ensure accuracy. Statistical significance was determined using a two-tailed p-value, with a threshold set at p < 0.05. It is worth noting that the DerSimonian and Laird method assumes that studies are estimating different but related intervention effects. This assumption aligns well with the heterogeneous set of observational studies included in our meta-analysis, thereby substantiating our methodological choices.

Results

Dyslipidemia Among Patients With Psoriasis and Control Subjects

**Figure 2:**
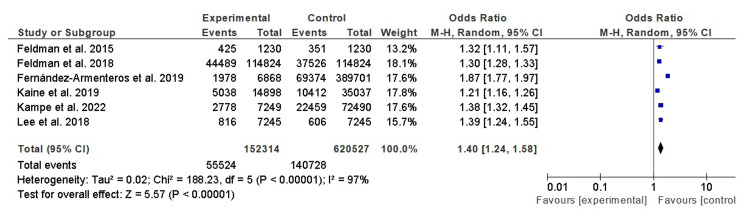
Dyslipidemia among patients with psoriasis and control subjects Forest plot illustrating the odds ratios (ORs) for dyslipidemia prevalence among patients with psoriasis compared to control subjects. Included studies: Feldman et al. (2015) [12], Feldman et al. (2018) [13], Fernández-Armenteros et al. (2019) [14], Kaine et al. (2019) [15], Kampe et al. (2022) [16], and Lee et al. (2018) [17].

Table 1 presents a comprehensive overview of findings from seven distinct studies examining the relationship between psoriasis and dyslipidemia. Each study is meticulously detailed, including the names of the authors, the methodologies employed, participant counts within the psoriasis and control groups, and the resulting outcomes. Crucially, the table furnishes ORs and 95% CIs, serving as quantifiable indicators of the strength of the association between psoriasis and dyslipidemia in each study. The collective evidence from these studies unequivocally demonstrates a statistically significant association between psoriasis and dyslipidemia. This is underscored by the overarching OR of 1.40 (95% CI, 1.24-1.58), signifying a 1.40-fold increased likelihood of dyslipidemia in individuals with psoriasis compared to their counterparts without the condition. The marked heterogeneity among these studies (I² = 97%, p < 0.001) highlights the diverse characteristics of study populations and methodological approaches, underlining the robustness of the collective findings. Figure 2 complements Table 1 by graphically presenting the ORs and 95% CIs for each of the included studies. These figures serve to substantiate the statistical significance and overall effect size of the association between psoriasis and dyslipidemia, further reinforcing the validity of the findings.

**Table 1:** Dyslipidemia among patients with psoriasis and control subjects

Authors	Methods	Psoriasis cases	Controls	Results
Feldman et al. (2015) [12]	Retrospective	425/6,868	351/1,230	Significant, USA
Feldman et al. (2018) [13]	Retrospective	44,489/114,824	37,526/114,824	Significant, USA
Fernández-Armenteros et al. (2019) [14]	Cross-sectional	1,978/6,868	69,374/398,701	Significant, Spain
Kaine et al. (2019) [15]	Retrospective	5,038/14,898	10,412/35,037	Significant, USA
Kampe et al. (2022) [16]	Cross-sectional	2,778/7,249	22,459/72,490	Significant, Slovakia
Lee et al. (2018) [17]	Retrospective	816/7,245	606/7245	Significant, USA

Obesity Among Patients With Psoriasis and Control Subjects

**Figure 3:**
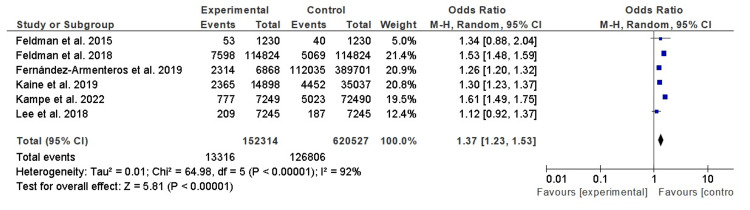
Obesity among patients with psoriasis and control subjects Forest plot showing the odds ratios (ORs) indicating the association between obesity and psoriasis severity. Included studies: Feldman et al. (2015) [12], Feldman et al. (2018) [13], Fernández-Armenteros et al. (2019) [14], Kaine et al. (2019) [15], Kampe et al. (2022) [16], and Lee et al. (2018) [17].

Table 2 closely mirrors the structure of Table 1, furnishing a comprehensive summary of findings from studies investigating the association between psoriasis and obesity. With careful attention to detail, this table elucidates the study authors, research methodologies, participant counts in both psoriasis and control cohorts, and the resultant outcomes. The ORs and 95% CIs are thoughtfully provided, offering quantifiable insights into the strength of the association between psoriasis and obesity in each study. The cumulative findings unambiguously affirm a significant link between psoriasis and obesity, underscored by an overarching OR of 1.37 (95% CI, 1.23-1.53). This signifies that individuals with psoriasis exhibit a 1.37-fold greater likelihood of being obese compared to their counterparts without the condition. Additionally, substantial heterogeneity is evident among the studies (I² = 92%, p < 0.001), underscoring the diversity in study populations and methodologies. Figure 3 visually encapsulates the OR and 95% CI for each of the studies investigating the relationship between psoriasis and obesity. These graphical depictions serve to enhance the comprehensibility and credibility of the findings.

**Table 2:** Obesity among patients with psoriasis and control subjects

Author	Methods	Psoriasis cases	Controls	Results
Feldman et al. (2015) [12]	Retrospective	53/1,230	40/1,230	Significant, USA
Feldman et al. (2018) [13]	Retrospective	7,598/114,824	5,069/114,824	Significant, USA
Fernández-Armenteros et al. (2019) [14]	Cross-sectional	2,314/6,868	112,035/398,701	Significant, Spain
Kaine et al. (2019) [15]	Retrospective	2,365/14,898	4,452/35,037	Significant, USA
Kampe et al. (2022) [16]	Cross-sectional	777/7,249	5,023/72,490	Significant, Slovakia
Lee et al. (2018) [17]	Retrospective	209/7,245	187/7,245	Significant, USA

Quality Assessment of the Included Studies

In our systematic review and meta-analysis, the quality assessment of the included studies was performed using the Newcastle-Ottawa Scale (NOS), a widely recognized tool for evaluating the methodological quality of non-randomized studies. The NOS criteria, which include selection, compatibility, and outcome domains, allowed us to assess each study's risk of bias. Table 3 presents the results of this assessment for each of the seven studies included in our analysis. Notably, Feldman et al. [13], Fernández-Armenteros et al. [14], and Feldman et al. (2015) [12] achieved higher overall scores, reflecting relatively high methodological quality. Conversely, Kaine et al. [15], Kampe et al. [16], and Lee et al. [17] exhibited moderate overall quality scores. These quality assessments are crucial in interpreting the robustness of our findings and understanding the potential impact of bias on our results.

**Table 3:** Newcastle-Ottawa quality assessment of the included studies

Reference	Selection (Max: 4)	Compatibility (Max: 2)	Outcome (Max: 3)	Overall (Max: 9)	Interpretation
Feldman et al. (2015) [12]	4	1	2	7	Moderate Quality
Feldman et al. (2018) [13]	3	2	2	8	Good Quality
Fernández-Armenteros et al. (2019) [14]	4	2	2	8	Good Quality
Kaine et al. (2019) [15]	3	2	2	7	Moderate Quality
Kampe et al. (2022) [16]	3	2	2	7	Moderate Quality
Lee et al. (2018) [17]	3	2	2	7	Moderate Quality

Discussion

In this comprehensive meta-analysis, we elucidated a significant association between psoriasis and two pivotal health concerns: dyslipidemia and obesity. Our findings revealed an OR of 1.40 (95% CI, 1.24-1.58) for the relationship between psoriasis and dyslipidemia and an OR of 1.37 (95% CI, 1.23-1.53) for the association between psoriasis and obesity. These results align with the meta-analysis conducted by Choudhary et al. in 2020, which reported similar findings, reinforcing the robustness and consistency of this association [18].

Our study's results also corroborate the findings of Miller et al. in 2013, who highlighted a robust link between psoriasis, obesity, dyslipidemia, and other components of the metabolic syndrome [19]. Intriguingly, our analysis unveiled that a high body mass index and female sex emerged as strong predictors of biological treatment discontinuation, supporting the observations made by Mourad et al. in 2019 [20]. This underscores the clinical significance of screening for obesity as an effective tool not only for optimizing treatment but also for promoting treatment adherence among individuals with psoriasis.

The complex interplay between psoriasis and obesity may find its roots in the disrupted function of the skin barrier and the lymphatic system [21-23]. Furthermore, the secretion of proinflammatory cytokines, such as tumor necrosis factor and interleukin-6, plays a pivotal role in mediating the inflammatory response observed in both conditions [24,25]. Notably, elevated levels of leptin have been identified as significant factors contributing to keratinocyte proliferation, while adiponectin has demonstrated anti-inflammatory properties, linking adipokines to psoriasis pathogenesis [26,27]. These insights suggest that targeting cytokine levels may hold promise as an effective preventive and treatment strategy for individuals with psoriasis.

Moreover, the adoption of healthy lifestyles among obese patients with psoriasis cannot be underestimated, as weight loss has been found to reduce both psoriasis skin lesions and associated joint disease [28]. Importantly, recent research has highlighted the efficacy of glucagon-like peptide-1 (GLP-1) agonists in reducing the psoriasis area and severity index and fasting plasma glucose in patients with psoriasis and diabetes mellitus [29].

Therefore, GLP-1 agonists may offer a valuable therapeutic option for patients contending with psoriasis, high body mass index, and diabetes.

The association between psoriasis and dyslipidemia raises concerns about the impact of psoriasis medications on lipid profiles. Cyclosporine and acitretin, commonly used in psoriasis treatment, have been linked to adverse lipid changes. Cyclosporine may disrupt lipid metabolism by inhibiting bile acid excretion and hepatic low-density lipoprotein (LDL) receptor expression, leading to increased LDL and decreased HDL levels. Acitretin can affect lipid synthesis and breakdown in the liver, contributing to dyslipidemia [30,31]. Given these risks, the regular monitoring of serum lipids and body mass index is crucial in psoriasis patients to prevent cardiovascular complications and improve treatment adherence. Furthermore, considering the potential of statins in addressing these medication-induced lipid changes is vital. Statins, known for their lipid-lowering and anti-inflammatory properties, could offer dual benefits in managing both the dyslipidemia and the systemic inflammation associated with psoriasis. Recent animal studies have also highlighted the importance of treating obesity and dyslipidemia to prevent exacerbations in psoriasis, suggesting a possible role for statins in this context [32]. However, the application of statins in psoriasis patients, especially those on medications like cyclosporine and acitretin, warrants careful consideration due to potential drug interactions and the need for further research to establish long-term safety and efficacy.

Intriguingly, recent research has shed light on the genetic underpinnings of psoriasis, particularly among young females, where high-density lipoprotein/triglyceride levels have been identified as genetic predictors of psoriasis susceptibility [21]. This adds a compelling dimension to our understanding of the intricate relationships between psoriasis, lipid metabolism, and genetics.

Despite these insights, our study has inherent limitations, primarily stemming from the relatively small number of included studies and their observational nature. Moreover, substantial heterogeneity was evident among the studies, reflecting the inherent variations in study populations and methodologies. These limitations emphasize the need for additional well-designed research to further elucidate the complex interplay between psoriasis, dyslipidemia, and obesity and the potential clinical implications for patient care.

## Conclusions

This meta-analysis provides compelling evidence supporting the intricate relationships between psoriasis, dyslipidemia, and obesity. Our comprehensive review of six studies consistently reveals significant associations between psoriasis and both dyslipidemia and obesity, emphasizing the systemic nature of psoriasis and its far-reaching health implications. Individuals with psoriasis face a substantially increased risk of developing dyslipidemia and obesity compared to those without the condition, with ORs of 1.40 and 1.37, respectively. These findings underscore the importance of holistic patient care, recognizing that psoriasis extends beyond dermatological manifestations and requires a multidisciplinary approach to address associated comorbidities. These results hold clinical significance, urging healthcare providers to consider the broader health implications of psoriasis and the need for early screening, prevention, and intervention strategies for dyslipidemia and obesity in affected individuals. While this meta-analysis enhances our understanding of the relationships among psoriasis, dyslipidemia, and obesity, further research is warranted to explore the mechanisms underlying these associations and to develop tailored therapeutic approaches. In summary, this study contributes valuable insights to the field, emphasizing the imperative of holistic care in managing psoriasis and promoting the overall well-being of affected individuals.
